# Eating behaviour and weight development of European and Asian seafarers during stay on board and at home

**DOI:** 10.1186/s12995-021-00329-9

**Published:** 2021-09-14

**Authors:** Felix Alexander Neumann, Lukas Belz, Dorothee Dengler, Volker Harth, Thomas von Münster, Joachim Westenhöfer, Marcus Oldenburg, Birgit-Christiane Zyriax

**Affiliations:** 1 Midwifery Science - Health Services Research and Prevention, Institute for Health Services Research in Dermatology and Nursing (IVDP), University Medical Center Hamburg-Eppendorf (UKE), Martinistraße 52, N26, 20246 Hamburg, Germany; 2 Maritime Medicine, Institute for Occupational and Maritime Medicine Hamburg (ZfAM), University Medical Center Hamburg-Eppendorf (UKE), Hamburg, Germany; 3 Competence Center Health, Faculty of Life Sciences, University of Applied Sciences (HAW), Hamburg, Germany

**Keywords:** Seafarer, Filipino, Burmese, European, Nutrition, Eating habits, Body weight, Anthropometrics

## Abstract

**Background:**

Food choices on board merchant ships are limited and seafarers repeatedly described as being at high risk of developing overweight compared to the general population. Up to date, research has not distinguished whether seafarers gain weight on board or at home and whether eating habits differ in both settings.

**Methods:**

As part of the e-healthy ship project, cross-sectional data were collected in two different measurements. In the first investigation on board of three merchant ships of German shipping companies, differences in eating behaviour at home compared to on board ships were assessed for 18 Burmese, 26 Filipino and 20 European seafarers. In a second study, BMI, weight development and location of body weight change of 543 Filipino and 277 European seafarers were examined using an online questionnaire on 68 ships.

**Results:**

According to the board examinations, foods and beverages consumed on merchant ships varied widely from seafarers’ diets in their home country. Burmese, Filipino and European seafarers equally reported to consume more fruit (z = 4.95, *p* < .001, *r =* .62) and vegetables (z = 6.21, *p <* .001, *r =* .79), but less coke (z = −5.00, *p <* .001, *r =* .76) when at home. Furthermore, culturally different changes were found across all other foods and beverages. The online questionnaire revealed that 45.8% of seafarers were overweight (55.4% Europeans vs. 40.8% Filipinos, *p* < .001) and 9.8% obese. Moreover, a higher percentage of Europeans compared to Filipinos reported weight gain over the course of their professional career (50.2% vs. 40.7%, *p* = .007). A sub-analysis of seafarers with weight gain found that more Europeans than Filipinos gained weight at home (43.9% vs. 23.1%, *p* < .001).

**Conclusions:**

Both, home and working on board merchant ships, represent very different living environments which may affect seafarers’ lifestyle and eating habits in various ways and thus could favour or inhibit weight gain. From our results, it appears that the body weight and eating habits of Asian seafarers in particular are adversely affected by the working and living conditions on board. Further prospective studies are required to prove this hypothesis.

## Introduction

Seafarers on merchant ships are at higher risk for overweight (body mass index [BMI] > 25 kg/m^2^) and cardiovascular disease than the general population [1–3]. The working and living conditions on board have repeatedly been described as a major influencing factor [4]. Over contract durations of 3–6 months for officers and 6–9 months for ratings [4], seafarers’ life on board is characterised by high levels of psychological and physical stress, limited opportunities for leisure activities and a restricted food choice [5]. In particular, the provision of food on board relies on a variety of factors. The time intervals between food deliveries and thus also the availability of fresh food depend on when the ships arrive at the port of supply [6]. The choice and quantity of food ordered is determined in consultation between the cook and the master and is limited by food budget of the shipping companies [7, 8]. Individual wishes are not taken into account. In addition, a shop provides access to high-fat snacks and sweets on board, whereas short stays in ports prevent self-supply on land. While the cook decides what dishes are offered on board, the choice of what to eat is the seafarers’ only way to influence their own diet. This complex nutritional situation on board leads to an unfavourable diet, which promotes the development of diseases [9, 10].

Results of a study on merchant ships of a German shipping company show that Kiribati seafarers ingest more calories from food on board (3315 kcal vs. 3094 kcal, *p* = .017) and had a higher BMI (30.1 kg/m^2^ vs. 25.4 kg/m^2^, *p* < .001) compared to their European colleagues [11]. Furthermore, it was found that eating habits and attitudes of seafarers from different cultural backgrounds were different which in turn was attributed to different dietary patterns on board [12]. However, these results did not offer any information about the seafarers’ nutrition during home stay – outside of their occupational context. Consequently, researchers have not yet been able to confirm whether or not poor eating behaviours at sea improved when a seafarer returned home. In addition, it has not yet been investigated, whether the weight was gained at sea or at home. Since seafarers are always in transition between the two living environments, it might be important for the planning of health-promoting measures to consider aspects of nutritional behaviour and weight development that occur outside the ship.

In general, little research has been done on the eating behaviour of shipboard employees from great seafarer nations such as those in Asia [8, 10]. This research gap needs to be closed as approximately 20% of the 1.2 million seafarers worldwide come from the Philippines [13] and about 4% from Myanmar [14].

Therefore, the present study aims to compare whether and to what extent the frequency of food and beverage groups consumed on board and at home differs among Burmese, Filipino and European seafarers. Furthermore, the nutritional status and weight development of Filipino and European seafarers was investigated and whether weight gain and loss occur on board or in the home country.

## Methods

E-healthy ship is an interdisciplinary, EU-funded project to improve health and health management of seafarers on merchant ships.

### Design and recruitment

This pilot study presents cross-sectional data obtained from two different measurements (Table 1). Between May and September 2018, questionnaires and standardised interviews were conducted by a nutritionist during sea voyages on board of two container ships (*n* = 24 each) and one bulk carrier (*n* = 26) of two German shipping companies (study I). The purpose and procedures of the study were presented to the crew. Every seafarer on board was asked to voluntarily participate by signing the consent form and was informed about the possibility to cancel participation at any time without giving reasons. There were no exclusion criteria in the selection of study participants.

**Table 1:** Study characteristics

Characteristics	Study I (questionnaires and examinations on board)	Study II (questionnaire sent to ships)
Time period	May–September 2018	February 2019
Number of ships	3	68
Number of participants	64	820
Nationalities included for analysis	Europeans, Filipinos, Burmese	Europeans, Filipinos

For the second study, a questionnaire was conducted on 68 ships of the two cooperating shipping companies in February 2019 (study II). Both studies obtained ethical consent from the Ethics Committee of the Medical Association of Hamburg and were conducted according to the Declaration of Helsinki.

### Data collection

Demographic data assessed for both study samples included nationality, age, occupational rank and the working area on the ship.

#### Study I

Information about body height was taken from the seafarers’ medical records, and body weight was measured by using a calibrated weighing scale from Kern®. As ship movements might impact the results of the examination, weight measurements were conducted when ships were anchored at port. Differences in food and beverage intake at home and on board were assessed in a semi-structured interview based on a self-developed questionnaire. In a controllable interview, language barriers and cultural differences within a multicultural team can be taken into account more effectively [15]. Moreover, the nutritionist as well as the responding seafarer were able to ask about any uncertainties, which thus ensured high response quality and increased data validity. Seafarers were asked to rate how often foods and beverages were consumed on average at home compared to at sea. Rated food groups were: *bread; rice; noodles; potatoes; vegetables; salad; fruits; milk & milk products; cheese; meat; sausage; fish; eggs; cakes, sweets, cookies & confectionery; chips & salted nuts.* Rated beverages were: *water; coffee; tea; coke; lemonade; iced tea; sweetened tea; fruit juice; beer & wine; spirits.* Responses were given on a standardised ordinal scale (“not part of my diet” = missing, “considerately less” = −2, “somewhat less” = −1, “equal” = 0, “somewhat more” = 1, “considerately more” = 2). Cronbach’s α was determined as a measure of the internal consistency of the self-developed study instruments. Internal consistency was acceptable for the food group scale (Cronbach’s α = .728) and good for the beverage scale (Cronbach’s α = .811).

#### Study II

The online questionnaire was sent to 68 ships of the two cooperating shipping companies via email and distributed to the crews by the ship officers. To ensure anonymity, the completed questionnaires were directly returned to the researcher team. Since we aimed to conduct a survey that was representative across all ranks of crewmembers, and thus the entire seafaring population of the shipping companies, no sampling strategies or exclusion criteria were applied. Seafarers were asked for demographical data, self-reported body height, self-reported body weight and whether body weight had increased, decreased or remained stable over the course of the professional career. Furthermore, the body weight of the seafarers at the age of 20 was queried. This made it possible to verify if self-reported weight data and weight development data correspond, which improved validity and reliability of the data. In a follow-up question, those seafarers who reported weight gain or weight loss were asked whether the change in body weight was more likely to have occurred at sea, at home or equally.

### Statistical analysis

Means and standard deviations were calculated to report demographic characteristics and one-way analyses of variance (ANOVAs) and t-tests used to compare mean values. Categorical variables were presented as percentages and differences examined by using chi-squared tests or Fisher’s exact tests for small numbers of cases. Post hoc testing was performed based on adjusted standardised residuals and post hoc *p*-values were adjusted according to the Bonferroni correction [16].

The ordinal data regarding food and beverage intake in study I were analysed by using multiple one-sample Wilcoxon signed-rank tests. The test value = 0 corresponds to the answer that the intake of the respective food or beverage did not differ between at home and on board. Significant results to a positive z-test statistic indicate that the food or beverage was consumed more frequently at home, while negative z-test statistics indicate an increased consumption on board. For the effect size, the z-test statistics was used to compute Pearson’s standardised regression coefficient r [17]: $r=\frac{Z}{\sqrt{n}}$. The values ​​were interpreted according to the categories *very low* (< .2)*, low* (.2 < .4)*, moderate* (.4 < .6)*, strong* (.6 < .8) *and very strong* (.8 ≤) as suggested by Bortz [18].

The calculation of the BMI was performed according to the World Health Organization [19]: BMI = (body weight in kg) / (body height in m)^2^. Overweight was defined as BMI ≥ 25 kg/m^2^, obesity as BMI ≥ 30 kg/m^2^. All statistical analyses were carried out with SPSS Statistics version 25 (IBM, Armonk, NY) and Microsoft Excel 2013 (Microsoft, Redmond, WA). The significance level was set to alpha = .05.

## Results

### Demographic and occupational characteristics of study I

Sixty-five out of 74 seafarers (86.5%) of the all-male crew on board of the three examined ships participated in study I. While one seafarer did not take part in the study for personal reasons, three other seafarers were so demanded by work during the study period that they did not have the opportunity to participate. Six other seafarers were part of a crew change shortly after the boarding of the research team and thus did not complete the survey.

The total study group consisted of 18 Burmese, 26 Filipinos, 20 Europeans and one Ethiopian seafarer. The latter was excluded from analyses as groups of one participant cannot be statistically compared. The European group consisted of seafarers from Ukraine (8), Lithuania (2), Poland (2), Romania (2), Russia (2), Hungary (1), Germany (1), Montenegro (1) and Slovakia (1). Average age, average BMI and the percentage distribution for BMI groups did not significantly differ between Burmese, Filipinos and Europeans (Table 2). The average BMI ranged between 25.9–26.3 kg/m^2^ and was overweight among all groups.

**Table 2:** Baseline characteristics for the measurements on board

Characteristics	Burmese (***n*** = 18)	Filipinos (***n*** = 26)	Europeans (***n*** = 20)	Test statistics	***p***-value
Age (years)	42.3 (±9.3)	39.6 (±7.1)	36.7 (±12.6)	1.63	.205^a^
BMI (kg/m^2^)	26.3 (±2.8)	26.3 (±3.9)	25.9 (±3.5)	0.09	.915^a^
BMI groups (%)				4.24	.392^b^
Normal weight	38.9	50.0	40.0		
Overweight	55.6	30.8	50.0		
Obesity	5.6	19.2	10.0		

*BMI* body mass index

Values are given as percentage or mean (standard deviation)

^a^one-way ANOVA

^b^Fisher’s exact test

### Differences in food and beverage intake at home compared to on board

Table 3 presents data comparing the frequency of foods and beverages consumed at home to at sea. The changes of food intake among Burmese, Filipino and European seafarers were mostly different, although some similarities can be found. The seafarers of all three groups stated that they consume more vegetables and fruits, but fewer coke at home than on board. Burmese and Filipinos also reported to ingest less *bread, cheese, sausage, cakes, sweets, cookies & confectionery, coffee* and *iced tea* in their homeland, but more *fish*. Filipinos and Europeans equally stated that they eat less noodles and egg at home than on board. While Burmese seafarers reported to eat more rice and salad at home; the Filipino seafarer’s average intake at home was lower for *potatoes, milk & milk products, meat, chips & salted nuts, tea, lemonade, beer & wine* as well as *spirits* and higher for *water*. Finally, the results for the European seafarers indicated lower intake of *rice* at home, but a higher consumption of *milk & milk products, cheese, cakes, sweets, cookies & confectionery*, *fruit juice* and *beer & wine*.

**Table 3:** Results of the one-sample Wilcoxon signed-rank test (*N* = 64; test value = 0): Seafarers’ consumption of foods and beverages at home compared to on board by nationalities

Foods and beverages	Burmese^**a**^					Filipinos^**b**^					Europeans^**c**^					Total^**d**^				
	n	Med	Z	p	r	n	Med	Z	p	r	n	Med	Z	p	r	n	Med	Z	p	r
**Foods**																				
Bread	13	–1	−3.02	.003*	**.84**	25	0	−2.94	.003*	.59	18	.5	1.38	.166	.33	56	0	−2.96	.003*	.40
Rice	18	.5	2.18	.030*	.51	25	0	0.37	.713	.07	19	−1	−3.39	.001*	**.78**	62	0	−0.73	.466	.09
Noodles	17	−1	−1.47	.140	.36	24	−2	−3.79	< .001*	**.77**	18	−.5	− 2.08	.038*	.49	59	−1	−4.43	< .001*	.58
Potatoes	18	0	−1.20	.229	.28	21	−1	−3.54	< .001*	**.77**	20	0	0.00	1.000	.00	59	−1	−2.85	.004*	.37
Vegetables	18	2	3.64	< .001*	**.86**	25	2	4.24	< .001*	**.85**	19	1	2.84	.005*	**.65**	62	1	6.21	< .001*	**.79**
Salad	17	1	2.86	.004*	**.69**	24	0	−0.90	.366	.18	20	1	1.79	.073	.40	61	0	1.93	.054	.25
Fruits	18	1	2.98	.003*	**.70**	26	1	2.79	.005*	.55	20	1	2.88	.004*	**.64**	64	1	4.95	< .001*	**.62**
Milk & milk products	18	0	0.87	.386	.20	25	0	−1.98	.047*	.40	20	1	3.02	.003*	**.68**	63	0	1.30	.193	.16
Cheese	12	−2	−3.04	.002*	**.88**	24	−1	−4.04	< .001*	**.82**	19	1	2.95	.003*	**.68**	55	−1	−2.26	.024*	.30
Meat	18	0	0.29	.773	.07	26	−1	−2.70	.007*	.53	20	0	−0.26	.793	.06	64	0	−1.69	.091	.21
Sausage	13	−2	−2.92	.004*	**.81**	26	−1	−4.18	< .001*	**.82**	17	0	−1.01	.313	.24	56	−1	−4.89	< .001*	**.65**
Fish	18	1	2.72	.007*	**.64**	26	2	4.18	< .001*	**.82**	18	.5	1.33	.182	.31	62	1	5.07	< .001*	**.64**
Eggs	17	0	1.10	.273	.27	26	−1	−2.79	.005*	.55	20	−1	−2.20	.028*	.49	63	0	−2.55	.011*	.32
Cakes, sweets, cookies & confectionery	17	−1	−2.36	.018*	.57	25	−1	−4.05	< .001*	**.81**	17	1	2.83	.005*	**.69**	59	−1	−3.21	.001*	.42
Chips & salted nuts	18	0	−1.55	.120	.37	23	−1	−3.73	< .001*	**.78**	14	0	−0.73	.465	.20	55	−1	−3.84	< .001*	.52
**Beverages**																				
Water	18	0	0.92	.358	.22	26	1	2.92	.004*	.57	20	0	−1.73	.083	.39	64	0	2.30	.021*	.29
Coffee	17	−1	−2.48	.013*	**.60**	23	−1	−3.27	.001*	**.68**	18	0	−1.10	.273	.26	58	−1	−4.03	< .001*	.53
Tea	14	0	0.89	.372	.24	22	−1	−3.36	.001*	**.72**	20	0	1.73	.084	.39	56	0	−1.24	.216	.17
Coke	11	−1	−2.74	.006*	**.83**	21	−1	−3.73	< .001*	**.81**	11	−1	−2.01	.045*	**.61**	43	−1	−5.00	< .001*	**.76**
Lemonade	14	0	−1.16	.248	.31	26	−.5	−1.99	.046*	.39	11	−1	−0.55	.584	.17	51	0	−2.21	.027*	.31
Iced tea	12	−1	−2.46	.014*	**.71**	26	−.5	−2.25	.025*	.44	8	1	1.00	.317	.35	46	0	−2.64	.008*	.39
Fruit juice	18	0	−0.10	.922	.02	25	0	−1.49	.136	.30	19	0	2.08	.038*	.48	62	0	0.09	.930	.01
Beer & wine	11	0	0.00	1.000	.00	26	−1	−3.63	< .001*	**.71**	19	0	2.00	.046*	.46	56	0	−1.21	.225	.16
Spirits	12	−1	−1.46	.145	.42	24	−1	−3.84	< .001*	**.78**	11	0	1.41	.157	.43	47	−1	−3.41	.001*	.50

^a^*n* = 18

^b^*n* = 26

^c^*n* = 20

^d^*n* = 64

Question: Compared to onboard, how frequently do you consume the following foods/beverages, when you are in your home country?

The test value Med: “considerately less” = −2, “somewhat less” = −1, “equal” = 0, “somewhat more” = 1, “considerately more” = 2

The foods of the respective food group are either (1) more frequently consumed on board [Z < 0], (2) equally frequent consumed on board and at home [Z = 0], (3) more frequently consumed at home [Z > 0]

*p < .05; strong (|r| > .6) and very strong (|r| > .8) effect sizes according to Rosenthal [17] are in boldface

### Demographic and occupational characteristics of study II

For the second study, questionnaires were sent to 1005 seafarers of 68 merchant ships. Of the 970 crew members (mainly Filipinos and Europeans) that participated (96.5%), 81 participants were excluded due to missing data for nationality (37), weight development (32) and gender (12). Further 69 participants from Myanmar (30), Ethiopia (17), India (15), Sri Lanka (4), Turkey (1), China (1) and Georgia (1) were eliminated from analysis because their nationalities each had too few participants for a meaningful statistical comparison. Five female participants were excluded for the same reason. Data of 820 seafarers, 543 Filipinos and 277 Europeans – who stated that they were from Ukraine (75), Romania (67), Poland (59), Russia (26), Germany (13), Bulgaria (11), Lithuania (9), Estonia (4), Portugal (3), Montenegro (3), Slovakia (3), Europe (2), Hungary (1) and Croatia (1) – were finally included in further analyses (Table 4). The average age of European seafarers was 39.4 (±11.6) years and thus not significantly different from Filipino seafarers at 38.6 (±9.4) years. The average BMI and the percentage distribution for BMI groups differed significantly between nationalities. The average BMI was higher for European seafarers than for Filipinos (t = 4.03; *p* < .001). Post hoc testing showed that Europeans were more likely to be overweight than Filipinos (z = 15.2; *p <* .001), while Filipinos were more likely to be normal weight than Europeans (z = 19.5; *p <* .001).

**Table 4:** Baseline characteristics for the online questionnaire (*N* = 820)

Characteristics	Total	Filipinos (***n*** = 543)	Europeans (***n*** = 277)	Test statistic	***p***-value
Age (years)	38.7 (±10.3)	38.6 (±9.4)	39.4 (±11.6)	1.03	< .302^a^
BMI (kg/m^2^)	25.6 (±3.4)	25.3 (±3.5)	26.3 (±3.1)	4.03	< .001^a^
BMI groups (%)				19.7	< .001^b^
Normal weight	44.4	50.0	33.6		
Overweight	45.8	40.8	55.4		
Obesity	9.8	9.2	11.1		

*BMI* body mass index

Values are given as percentage or mean (standard deviation)

^a^t-test

^b^chi-squared test

### Weight development of seafarers

**Fig. 1:**
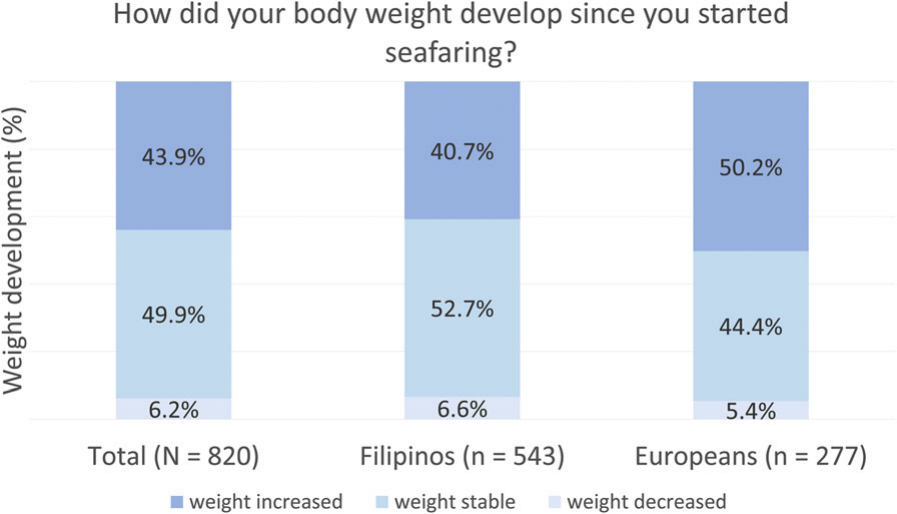
Body weight development since the start of seafaring. *Chi-squared test: χ2 = 6.80; p = .033*

The development of seafarers’ body weight over the course of their professional career was reported as increasing in 43.9%, decreasing in 6.2% and remaining the same in 49.9% (Fig. 1). A comparison of the seafarers’ nationality suggests a statistically significant difference in weight development (χ^2^ = 6.80, *p* = .033). Europeans were more likely to gain weight than Filipinos (50.2% vs. 40.7%; p_adj._ = .044). Pairwise comparisons of seafarers with stable weight (44.4% Europeans vs. 52.7% Filipinos; p_adj._ = .194) and reduced weight (5.4% Europeans vs. 6.6% Filipinos; p_adj._ = 1.000) did not reach statistical significance.

### Weight change can take place in both settings

**Fig. 2:**
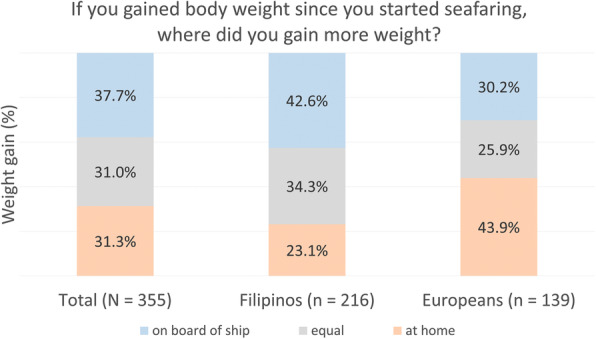
Place of body weight gain since the start of seafaring. *Chi-square*d *test: χ*^*2*^ *= 17.43. p < .001*

Seafarers who reported an increase of weight were asked for the setting where they had normally gained more weight (Fig. 2). While 37.7% responded to have gained more weight on board of ship, in 31.3%, the increase took place at home and for 31.0% equally at sea and at home. The pairwise comparison of nationalities indicated that the proportion of Europeans who gained weight at home was significantly greater than the proportion of Filipinos (43.9% Europeans vs. 23.1% Filipinos, p_adj._ < .001). Additionally, more Filipinos than Europeans reported weight gain on board (42.6% Filipinos vs. 30.2% Europeans, p_adj._ = .113) and in both locations equally (34.3% Filipinos vs. 25.9% Europeans p_adj._ = .578).

**Fig. 3:**
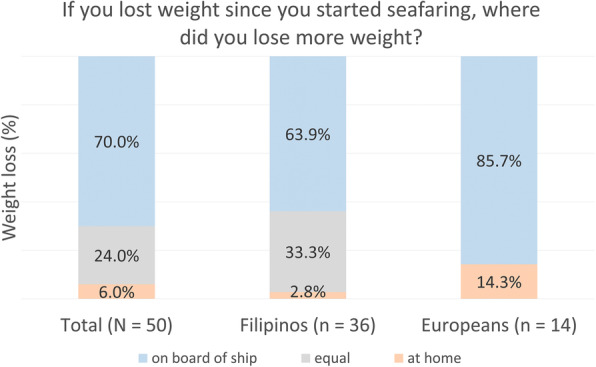
Place of body weight loss since the start of seafaring. Fisher’s exact test: χ^2^ = 7.58. *p* = .012

Seventy percent of seafarers with a decrease in weight (*n* = 50) reported to have lost more weight on board (85.7% Europeans vs. 63.9% Filipinos), 6% at home (14.3% Europeans vs. 2.8% Filipinos) and 24% in both places equally (0.0% Europeans vs. 33.3% Filipinos) (Fig. 3). Even though the distribution showed a significant result (χ^2^ = 17.43, *p* < .001), the pairwise comparisons did not indicate significant differences and thus could not determine which individual categories differed from each other.

## Discussion

To our knowledge, this study is the first one that compares food intake and the development of body weight of seafarers at home and on board of merchant ships. According to the on-board examinations, foods and beverages consumed on merchant ships differed widely from seafarers’ diets in their home country. Burmese, Filipino and European seafarers equally reported higher fruit and vegetable and lower coke consumption at home and several culturally different changes among all other food and beverage groups. Based on the self-reported data of the online questionnaire, 45.8% of seafarers were overweight and 9.8% obese. Furthermore, 43.9% stated to have gained body weight over the course of their professional career. While European seafarers were more likely to be overweight and gain weight compared to their Filipino counterparts, it could not be determined whether the increase in body weight was more likely to occur at home or aboard ships.

### Origin-related differences in BMI

When comparing the BMI of our study sample with the values of a corresponding general male population from a study of the NCD (non-communicable diseases) Risk Factor Collaboration [20], differences depending on the seafarers’ origin become apparent. More European seafarers are overweight (55.4% vs. 41.8%) but less obese (11.1% vs. 21.7%) than in the general Middle and Eastern European population. Furthermore, Filipino seafarers appear to be more likely to be overweight (30.8% vs. 21.6%) and obese (19.2% vs. 5.5%) compared to the Filipino general population. These figures are also reflected in average BMI, which is slightly lower for European seafarers (26.3 kg/m^2^ vs. 26.8 kg/m^2^) and higher for Filipino seafarers (25.3 kg/m^2^ vs. 22.9 kg/m^2^) compared to the respective general population. Thus, the occupational profile of the seafarer seems to have a greater influence on the BMI of Filipinos than of Europeans.

### The food supply on board influences dietary pattern

Our results show that the food and beverage intake of seafarers depends on the living and working environment. The overall shift in eating habits from “at home” to “on board” involves disadvantageous changes. For example, a high consumption of fruit and vegetables would be beneficial for health, as is a decreased intake of foods high in sodium, like sausage, as well as beverages high in sugar, like coke [21–23]. The actual supply of food on board is impacted by many factors. Food availability depends on the delivery capacity and possible shortages of caterers, the port of supply, restricted storage space on board and the limited budget provided by the shipping company [24]. These factors affect all seafarers identically, nevertheless, we found fewer significant differences in food intake between home and on board for European seafarers than for Burmese and Filipinos. Foods and beverages supplied on board seem to be more oriented towards European standards, indicating a mismatch between seafarers’ food preferences and food orders. A culture-related optimisation of food orders is recommended to ensure the provision of commonly consumed food groups and thus also a diverse and sufficient supply of nutrients. Employing crews with seafarers of one nationality would simplify the provision of a culture-specific nutrition and health-promoting conditions on board but is difficult to implement in practice.

### Seafarers’ overweight and weight development is more complex than overeating and unhealthy diet on board

So far, the nutritional status and weight gain have been attributed to frequent overeating and unhealthy diets among seafarers during stay on vessels [9, 24, 25]. On board of the merchant ships, meals are prepared by a cook and large portion sizes offered free of charge [25]. Hence catering on board represents a tempting eating environment which encourages overeating. This finding is consistent with the results for our study sample, as more than one-third of the seafarers with self-reported weight gain during their careers also reported gaining more body weight on board. However, it is further remarkable that for another third of the seafarers, weight gain was more likely to occur at home, suggesting that for more than two-thirds of seafarers, the environment plays a role in weight gain.

In general, overeating can be promoted by physical and psychological factors which might apply to seafarers as well, such as lack of sleep, poor hydration and stress [26–28]. According to the *Pavlovian behavioural conditioning*, the incentive of food increases and contributes to satisfaction, when the ingestion of foods is linked to a rewarding consequence [29]. The reward is an amplifying stimulus. As food is described as one of the few pleasures of the seafarers’ daily routine [7], cravings for food or eating can arise and thereby encourage overeating. In this context, food could also be understood as reward if the supply of culturally related food reminds seafarers of home.

An opposing example of overeating was described for Kiribati seafarers. For them, the westernised food environment on board represents something long desired, where food is offered that is not available in their home country [12]. This demonstrates that most importantly the seafarers must be satisfied with the food offered, so that cravings arise for daily meals on board [29]. Since 98.4% of cooks in study II were of Asian origin, it stands to reason that Filipino seafarers might be more satisfied with the meals offered than European seafarers. This goes along with the results of Zyriax et al. [9] who reported that more than 60% of the European seafarers would appreciate better trained cooks. The dissatisfaction with served meals on board could lead to overeating back home, which would explain why significantly more Europeans than Filipinos reported weight gain at home. Furthermore, the westernised calorie-rich food offered on board is not available for the Asian seafarers when staying at home. Finally, the change between both locations itself could also be another influencing factor for weight development. Seafarers might be at risk to adapt to a high energy intake on board and transfer this eating pattern to at home where the individual energy needs are lower.

### The impact of psychological and physical factors needs further investigation

Seventy percent of all seafarers who had lost weight, reported that weight loss occurred on board. It is likely that other factors than food intake contribute to weight change in seafarers as well. One of these factors certainly is the total daily energy expenditure. Considering the fact, that physically demanding work should support to maintain a normal body weight, it is almost surprising that large proportions of seafarers are overweight or obese [30]. Due to high levels of physical strain during working-hours, it is likely that seafarers expend more energy on board compared to their free-time at home which could favour weight loss on board. However, the amount of physical strain depends on the occupational profile of the seafarer and no in-depth studies have yet been carried out to investigate the differences in physical activity and energy consumption on board and at home. Also, psychological components caused by the work situation on board, such as social isolation and loneliness, were frequently described among seafarers [31] and could play an important role in eating habits and weight development [32, 33]. Furthermore, many seafarers work in shifts, which has been linked to overweight and obesity for other occupational groups [34]. To learn more about the underlying factors of seafarers’ weight development, more detailed tracking of body weight and further in-depth research on eating behaviour including psychological aspects are required.

### The cook at the Centre of a holistic nutrition intervention

Our results show the need for sustainable changes in the supply system on board merchant ships in order to make the catering on board more demand-oriented, culture-specific and overall healthier. By instrumentalising cooks as a *gatekeeper for nutrition*, Hjarnoe and Leppin [7] reported that promotion of a healthy diet at sea is possible but needs to overcome the occupational challenges of the maritime industry. Such changes are both large and small-scale and need to address not only the cook but also all other levels involved in maritime nutrition, such as legal guidelines as well as its implementation by the shipping companies. Practical methods for cooks to practice on board were already explored in an intervention study with ship cooks [7]. In addition, Westenhöfer et al. [12] suggested to use the principles of nudging, e.g., to offer fruits and vegetables as appetiser, as a promising strategy to influence seafarers’ food choices and consumption. However, when developing such an intervention, it should also be questioned how health promotion can support the seafarers to prevent the development of overweight at home.

### Limitations

Surveys on merchant ships are of course subject to limitations. The versatility of seafarers concerning origin, religion, culture, socioeconomic status, the different types of jobs and working conditions, as well as the prevailing living conditions on board merchant ships reflect a variety of difficulties for scientific investigations of seafarers’ nutrition and health. The merge of European seafarers of different countries as one group was necessary in order to enable evaluation, thus cultural differences among various European nationalities may bias the results for this group. Secondly, European seafarers were associated with the rank *officer* and Filipinos with *crew ranks*. Mixing of findings regarding cultural differences and socioeconomic status cannot be ignored along with genetic factors. Thirdly, since in study I the data of Burmese seafarers was exclusively gathered on the bulk carrier of one shipping company and the data of Filipino seafarers exclusively from container ships of another shipping company, it cannot be ruled out that differences between these groups arose due to the type of ship or shipping company guidelines. For study II, however, Filipino seafarers on merchant ships of all types were included in the survey. Fourthly, findings and conclusions are limited to male seafarers of the investigated nationalities. Fifthly, it needs to be mentioned that the small study population for study I divided into three groups only allowed finding significant differences in group comparisons that were at least of medium effect size. Small effect sizes remained statistically insignificant. For that reason, also a more detailed analysis, for example, among crew ranks, was not possible regarding the small dataset. Lastly, this study is based on a cross-sectional approach which excludes the possibility of cause-effect interpretations.

## Conclusion

Both, at home and aboard merchant ships, represent different living environments which seem to affect seafarers’ lifestyle and food intake in various ways and thus could favour or inhibit weight gain and weight loss. Thus, seafarers of different origins are likely to be influenced differently. From our results, it appears that the body weight and eating behaviour of Asian seafarers in particular are adversely affected by the situation on board within and outside the work context. Further prospective study is needed to prove this hypothesis and to add evidence as which underlying aspects are the main contributors to seafarers’ weight development.

## Data Availability

The datasets used and/or analysed during the current study are available from the corresponding author on reasonable request.
